# A 12–20 GHz Wideband High-Power SP2T Switch Based on Gap Waveguide Technology

**DOI:** 10.3390/s21165396

**Published:** 2021-08-10

**Authors:** Abdullah J. Alazemi, Ali Farahbakhsh, Davoud Zarifi

**Affiliations:** 1 Department of Electrical Engineering, Faculty of Engineering and Petroleum, Kuwait University, Kuwait 13060, Kuwait; 2 Electrical and Computer Department, Graduate University of Advanced Technology, Kerman 7631818356, Iran; a.farahbakhsh@kgut.ac.ir; 3 Electrical and Computer Engineering Department, University of Kashan, Kashan 8731753153, Iran; zarifi@kashanu.ac.ir

**Keywords:** microwave switch, SP2T switch, wideband devices, gap waveguide technology

## Abstract

A novel wideband high-power single-pole two-throw (SP2T) switch based on gap waveguide technology is presented in this article. The proposed switch has a SP2T structure and consists of three standard WR62 waveguide ports. Due to the advantage of gap waveguide technology, the switch design structure requires no electrical contact between its different parts, and the leakage of the electromagnetic wave is suppressed. The proposed switch has an air gap between its parts. As a result, the sliding part of the switch can be moved freely to change the switch states. Consequently, a low-precision and low-cost fabrication can be utilized. The simulation and measurement of the proposed switch indicate that a 50% operating frequency bandwidth covering the range of 12–20 GHz can be achieved. The switch input return-loss is better than 15 dB within the frequency bandwidth, whereas the insertion loss and isolation levels of the proposed design are above 0.15 dB and better than 65 dB, respectively.

## 1. Introduction

In recent years, the fast growth of technologies to enhance performance and reduce manufacturing cost has become a challenge in microwave engineering. Microwave switches, which are considered to be key components in many microwave and millimeter-wave circuits and systems, are widely utilized in radar systems, satellite transponders, communication links, microwave measurement equipment, and other systems. The main purposes of microwave switches are to provide redundancy in high-power radar systems and select filter banks, system calibration, and routing signals to different sub-systems [1,2,3,4,5].

There are many critical parameters that should be considered in designing microwave switches, such as obtaining low input return loss, low insertion loss, and high isolation levels. These also include achieving an acceptable switching time, excellent repeatability, high power handling, and long operating life. There are many articles on microwave switches that have been published recently and indicate that the microwave switch can either be a reflective, absorptive, or transfer switch [1]. In reflective switches, the electromagnetic (EM) wave is supposedly reflected to the input port when the switch is in the off state, whereas, in the absorptive switches, the EM wave must be dissipated. However, in transfer switches, the EM wave is routed to different outputs according to the switch states. 

Several microwave switches based on various technologies are also reported in the literature, including semiconductor devices such as PIN diodes and FETs [6]. These switches have advantages such as high switching speed, compact size, and low fabrication cost. However, they suffer from high insertion loss, low isolation between switch ports, and low power handling. RF micro-electromechanical systems (RF-MEMS) are also considered to be good candidates to design microwave switches [7,8,9,10]. The RF-MEMS structures have several advantages and features in both mechanical and solid-state technologies. However, their frequency bandwidth and maximum power handling are limited.

Mechanical waveguide switches are attractive and efficient in microwave frequency bands. They provide low insertion loss, good shielding with high isolation levels, and high-power handling capabilities [9,10,11]. Despite their excellent microwave performance, their complicated and expensive fabrication process is their main weakness since accurate and high precision technologies are required for manufacturing, especially at high-frequency bands. 

Gap wave technology has been proposed recently as an alternative solution to waveguide structure fabrication challenges [12,13,14]. According to EM theory, a PEC/PMC parallel plate structure has a bandgap when the spacing between two plates is smaller than a quarter wavelength, and this bandgap prevents EM wave propagation. Gap waveguide technology utilizes the bandgap as waveguide sidewalls to confine the EM wave to a particular path. Consequently, different metal layers of the structure do not need good electrical connections, and they can be placed on each other without any contact while EM wave leakage and unwanted radiation are suppressed. As a result, an isolated waveguide structure is obtained. Gap waveguide structures can be fabricated using various low-cost manufacturing technologies such as die sink electrical discharge, multilayer die pressing, electron beam melting, 3D printing, molding, and CNC milling. Therefore, the fabrication of these structures is affordable and cost-effective, particularly at high-frequency ranges. Accordingly, many millimeter-wave devices and applications are presented based on gap waveguide technology, such as planar array antennas [15,16,17,18], filters [19,20,21], amplifiers [22], couplers [23,24,25], phase shifters [26], and rotary joints [27]. 

In addition, gap waveguide technology is a great candidate for designing microwave switches, due to its contactless feature. In particular, a gap waveguide microwave switch is reported for Ku-band applications [28]. The proposed structure of the switch is complicated as it uses a cylindrical pin structure, and therefore, a complex manufacturing process is required to fabricate the switch. The proposed switch operating frequency bandwidth is narrow and limited between 10.5 and 13.5 GHz (25%). 

In this article, groove gap waveguide technology is utilized to design and develop a single-pole two-throw (SP2T) microwave switch with a simple planar structure and a 50% operating frequency bandwidth covering the range of 12–20 GHz. The proposed switch has wide bandwidth, low insertion loss, high isolation, and power handling capability for high power applications. The proposed switch can be used in radar systems, satellites, and any wideband Ku-band applications.

## 2. WR-62 to Groove Gap Waveguide Transition

**Figure 1:**
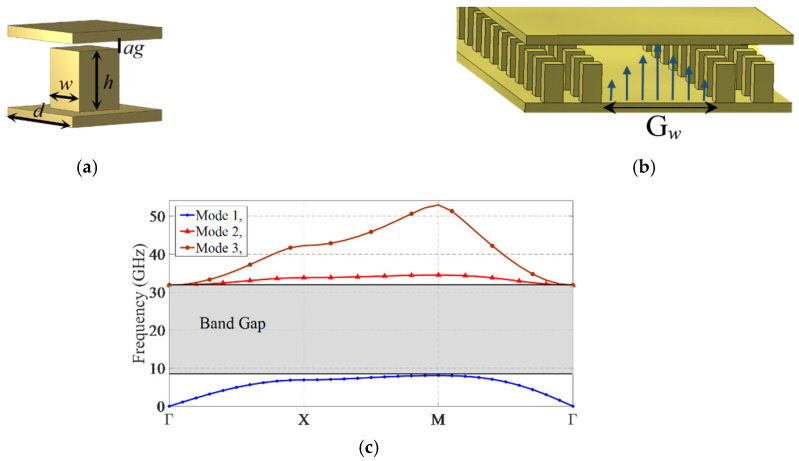
(**a**) Periodic pin structure, (**b**) groove gap waveguide structure, and (**c**) the dispersion diagram of the pins. The parameters are *h* = 4.5 mm, *w* = 1.65 mm, *d* = 4.1 mm, *ag* = 0.1 mm, and *G_w_* = 15.8 mm.

Figure 1 shows the geometry of the periodic pins and a groove gap waveguide (GGW) structure and dimensions, as well as the simulated dispersion diagram of the periodic pin structure. To realize the PMC surface, a periodic texture of metallic pins [15,16,17,18,19,20,21,22,23,24,25,26,27,28] or holes [29,30] can be used, and by placing a metallic plate on its top, the bandgap is achieved. The periodic pin structures are more compact and have a broader bandwidth compared to the holey structures. In contrast, the manufacturing of periodic holey structures is more cost-effective. In this paper, a periodic pin structure is utilized due to its compactness and wide bandwidth. However, the proposed switch can also be designed using a periodic holey structure. By selecting the proper dimensions for the pins, the frequency range of the bandgap is determined. Full-wave simulation of the structure is performed using the eigen-mode solver of the CST Microwave Studio. Figure 1c shows the bandgap of the periodic pins. The bandgap covers from 8 GHz to 32 GHz, which includes X-, Ku- and K-bands. It is observed that the electromagnetic wave cannot propagate along the periodic pin structure within the specified frequency band. By creating a groove between the pins, the wave can propagate along it.

**Figure 2:**
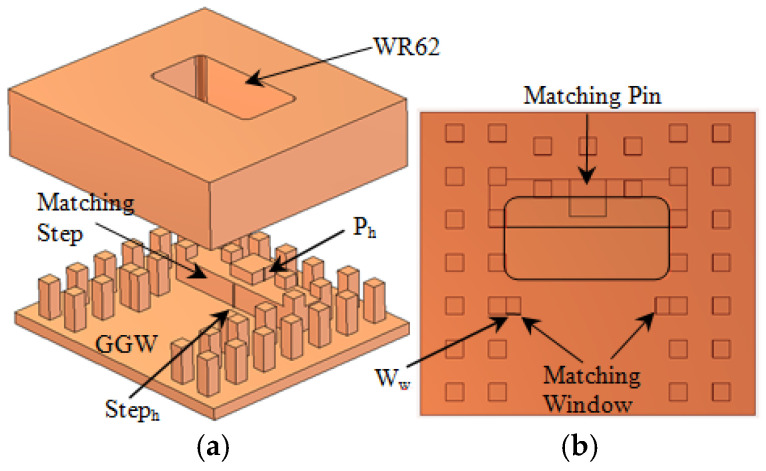
(**a**) Exploded view and (**b**) top view of the WR62 to GGW transition structure. The parameters are step height (Step_h_ = 3 mm), step width (Step_w_ = 4.5 mm), pin height (P_h_ = 1.5 mm), pin width (P_w_ = 3.5 mm), and window width (W_w_ = 1.3 mm).

The main part of the proposed microwave switch is a transition from standard WR62 waveguide to GGW, which is shown in Figure 2. The GGW is created using two rows of the periodic pins to achieve as high of an isolation level as often as possible. The WR62 standard waveguide is coupled to the GGW from its top layer. A metallic step is placed under the WR62 to rotate the EM waves and maximize the coupling between WR62 and GGW. To extend the bandwidth of the transition, two matching posts are introduced to the structure, i.e., a metallic matching pin and window as shown in Figure 2b. The metallic window has an inductive impedance, whereas the metallic pin acts as a capacitive impedance. Therefore, a wide impedance bandwidth can be obtained by selecting proper values for the pin and window dimensions as well as the metallic step. Briefly, the procedure of initial design of transition can be summarized as follows: 

Choose the height and width of the step as h/2 and λ0/4, respectively.

Choose the dimensions of the matching pin (P_w_ × P_w_ × P_h_) as 2w × 2w × h/2.

Choose the width of the inductive pins (w_w_) as w.

**Figure 3:**
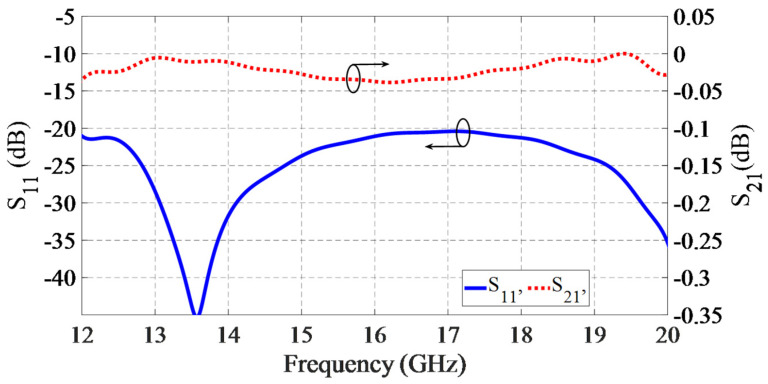
Simulated S-parameters of WR62 to GGW transition.

The optimized parameters are mentioned in the caption of Figure 2. The simulated S-parameters (S_11_ and S_21_) of the transition are shown in Figure 3. The transition structure is simulated using 3D EM simulation software (CST), and the embedded optimization tool (trust region framework) is utilized to tune the design parameters. The structure material is aluminum, in which the conductivity is 3.56 × 10^7^ S/m. The reflection coefficient of the transition is less than −20 dB in the desirable frequency bandwidth from 12–20 GHz, which shows that excellent impedance matching is obtained from the proposed structure. The insertion loss is less than 0.05 dB within the same frequency bandwidth, which proves the high efficiency of the transition and its excellent wave confinement inside the GGW structure.

**Figure 4:**
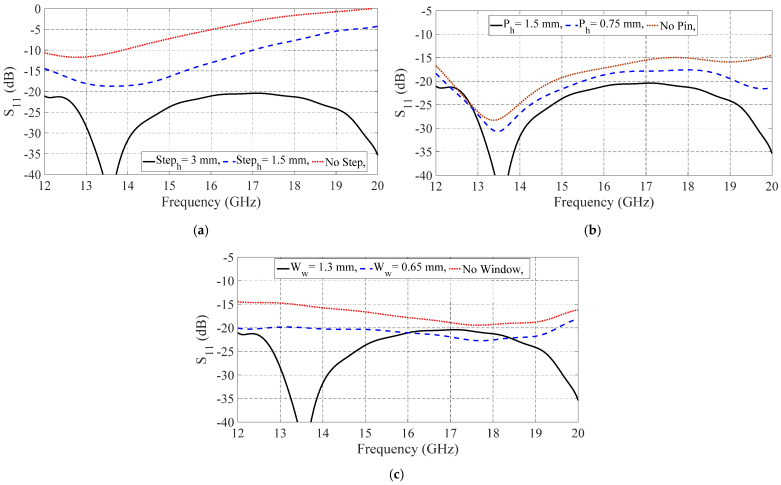
Simulated S-parameters of the transition by sweeping the design parameters: (**a**) step height, (**b**) pin height, and (**c**) window width.

According to Figure 4a, the step height has a significant effect on the wave rotation and coupling to the GGW. By removing the step, the parametric sweep analysis was performed on the step height (Step_h_), pin height (P_h_), and window width (W_w_) to investigate the effects of these parameters on the transition performance, as shown in Figure 4. According to Figure 4a, the step height has a significant effect on the wave rotation and coupling to the GGW. By removing the step, the input wave reflects back to the input port and cannot be coupled to the GGW. The effects of the pin height and window width on the bandwidth enhancement are apparent in Figure 4b,c.

## 3. Switch Design and Simulation Results

**Figure 5:**
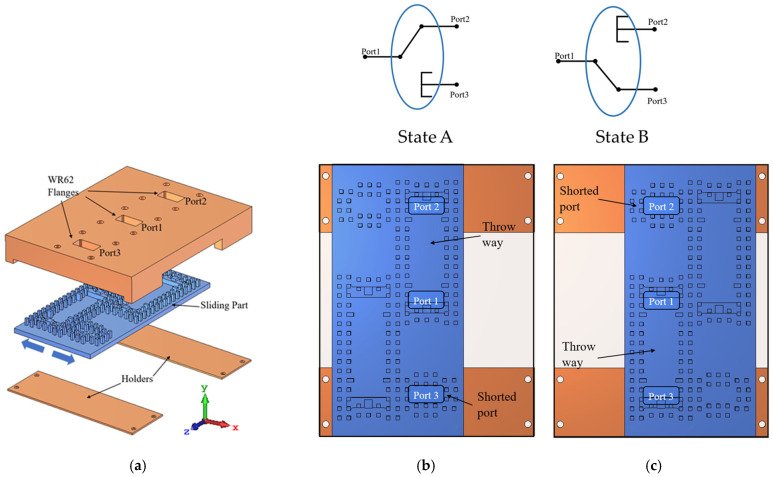
Geometry configuration of the proposed switch. (**a**) Exploded view and top view of (**b**) State A and (**c**) State B.

The proposed microwave switch is an SP2T based on GGW structure. The geometry of the proposed switch is shown in Figure 5. The input and output ports are located in the top layer. The middle layer, which is the switch sliding part, consists of all gap waveguide pins and matching posts. The switch state can be changed by sliding this layer to the left or right. In addition, two holder parts are introduced to the structure to provide mechanical stability to the sliding part, and they have no effect on the EM functionality of the switch. 

The switch mechanism works as follows: when the switch is in State A, the pole-port (Port 1) is coupled to the first throw-port (Port 2) using two transitions in a back-to-back arrangement. The EM waves are coupled from Port 1 to the GGW using the first transition, and then they are coupled to Port 2 using the second transition. In this state, Port 3 is shorted using a short-ended cavity in which the side walls are the periodic pins.

An inverted copy of the pins and posts is placed beside the first one to form State B as shown in Figure 5c. Due to the gap waveguide concept, the sliding part does not require any connection to the top layer. Consequently, it can slide freely while the wave leakage is totally suppressed by the gap waveguide structure. When the switch is in State B, the pole-port (Port 1) is connected to Port 3, whereas Port 2 is shorted.

**Figure 6:**
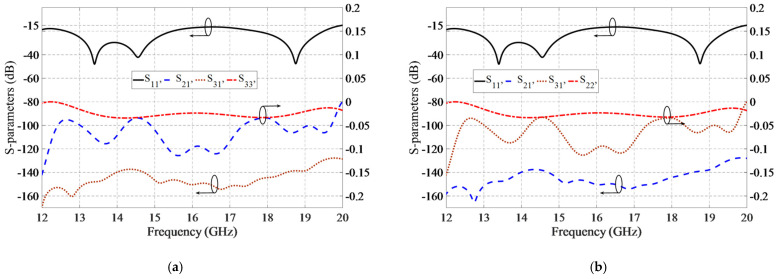
The simulated S-parameters of the microwave switch in (**a**) State A and (**b**) State B.

The simulated S-parameters’ results of the switch in states A and B are shown in Figure 6a,b, respectively. The reflection coefficient (S_11_) of the pole-port (Port 1) is less than −15 dB within the operating frequency bandwidth (12–20 GHz), which proves the excellent impedance matching of the switch. The insertion loss (S_21_) of the switch is less than 0.1 dB, and the isolation between ports 1 and 3 (S_31_) is better than 130 dB within the operating frequency bandwidth. The simulated results indicate that wave leakage is extremely low, and that the switch performance is excellent. The reflection coefficient (S_33_) of the shorted port is above −0.03 dB within the frequency bandwidth, which shows that the wave is totally reflected back, and the pins have no wave leakage. These specifications are due to the large bandwidth performance of the groove gap waveguides and the transitions from GGW to WR62.

**Figure 7:**
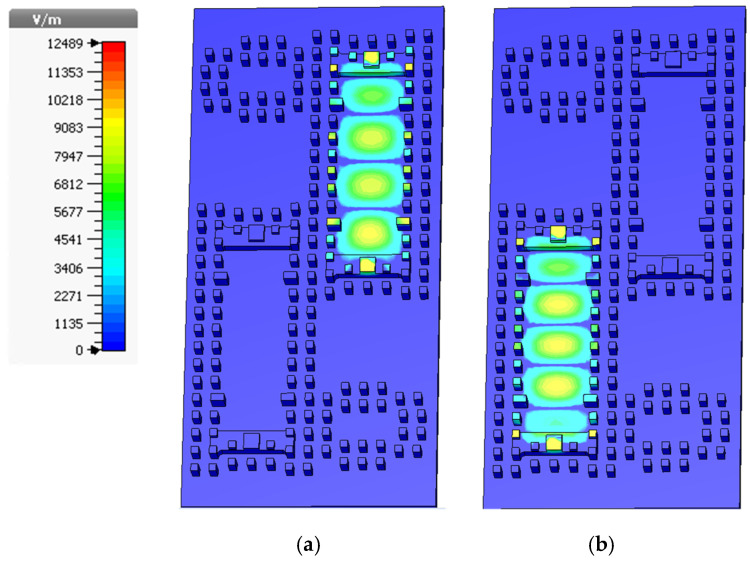
The simulated electric field distribution by excitation of the pole-port in (**a**) State A and (**b**) State B.

Due to the perfect symmetry of the structure for both States, A and B, the simulation results are the same in both states. The electric field distributions of the switch (by excitation of the pole-port) are also simulated for both states at the center frequency (16 GHz), illustrated in Figure 7. According to the figure, the EM waves are confined in the GGW and the proper coupling from the pole-port to Port 2 or Port 3 is apparent. To evaluate the power handling capacity of the proposed switch, the pole-port is excited by a 1-Watt EM wave power, and the maximum electric field in the structure is observed using a time-domain solver simulation in the CST. Figure 7 shows the electric field distribution in the structure for different states. Since the power is proportional to the square of the electric field intensity [31], the maximum power handling of the switch can be calculated as follows.

(1) $$P_{max}={\left(\frac{E_{d}\sqrt{P_{ext}}}{E_{max}}\right)}^{2}$$

where, *E_d_* is the air breakdown, *E_max_* is the maximum electric field intensity inside the switch, and *P_ext_* is the power of the excitation wave. As shown in Figure 7, *E_max_* is equal to 12,489 V/m. Since the air breakdown is *E_d_* = 3 MV/m and *P_ext_* = 1 W, the power handling capacity of the switch can reach up to *P_max_* = 57.7 KW.

**Figure 8:**
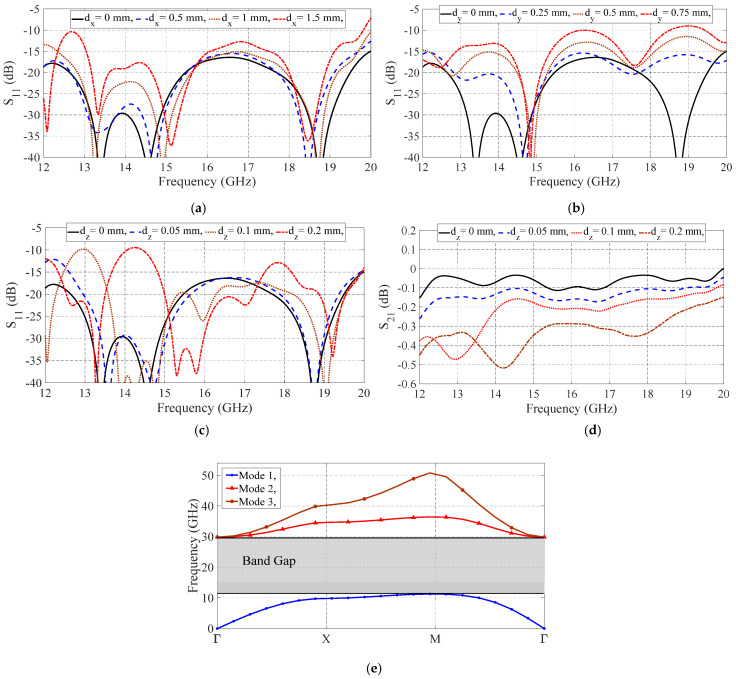
Simulated effects of the sliding part displacement in (**a**) X-axis, (**b**) Y-axis, (**c**) and (**d**) Z-axis on the performance of the switch, and (**e**) bandgap of the pins with *ag* = 0.3 mm.

In practical situations and due to mechanical tolerances, the position of the sliding layer may have slight displacements. Therefore, it is critical to investigate the effects of the sliding part displacement on switch performance. Accordingly, displacements in all directions are applied to the sliding part, and the input matching of the switch is simulated, shown in Figure 8. The switch performance is disturbed by distortions in different axes, but the performance is acceptable for a displacement of 1 mm on the X-axis, 0.5 mm on the Y-axis, and 0.1 mm on the Z-axis. The X-axis is the direction of the switch sliding, and Z-displacement (d_z_) is identical to the air gap changes. The simulated insertion loss of the switch for different d_z_ is plotted in Figure 8d.

As can be seen in Figure 8d, when d_z_ increases, the insertion loss aggravates, but it still is less than 0.5 dB, which is acceptable. In addition, the dispersion diagram of the period pins is simulated when the air gap is 0.3 mm (equal to 0.2 mm Z-axis displacement), shown in Figure 8e. Although the bandgap is decreased (11 GHz to 30 GHz), it covers the whole frequency bandwidth, and therefore, no wave leakage occurs. As a result, the switch is not too sensitive to the sliding part movements, and it can be manufactured using a low-cost method.

## 4. Fabrication and Measurement

**Figure 9:**
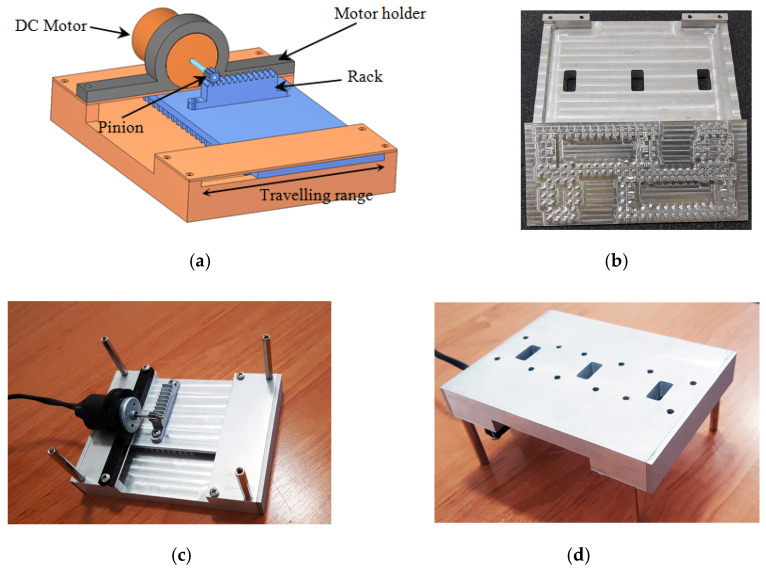
(**a**) Schematic of the switch with the linear actuator and photographs of the fabricated waveguide switch: (**b**) non-assembled, (**c**) assembled bottom view, and (**d**) assembled top view.

The proposed switch was manufactured using aluminum by a standard CNC milling technique in order to verify and test the design performance. Several photographs of the fabricated switch are shown in Figure 9. In order to change the switch state automatically by an electrical signal, a linear actuator was installed on the switch, which consisted of a DC motor and a rack and pinion structure as shown in Figure 9a. The pinion was connected to the motor shaft and engaged to the rack, which was attached to the sliding part of the switch. By clockwise or anticlockwise rotations of the motor shaft, the rack was moved to the right side or left side, and consequently, the switch state could be changed electrically. The traveling range of the sliding part was restricted by the metallic walls as depicted in Figure 9a. Thus, the motor moved the sliding part as far as possible to place the switch into the anticipated state. By current monitoring of the motor, it was detected that the sliding part reached the end of its traveling range, and then the motor turned off. The switching time of the switch is about 150 to 200 milliseconds.

**Figure 10:**
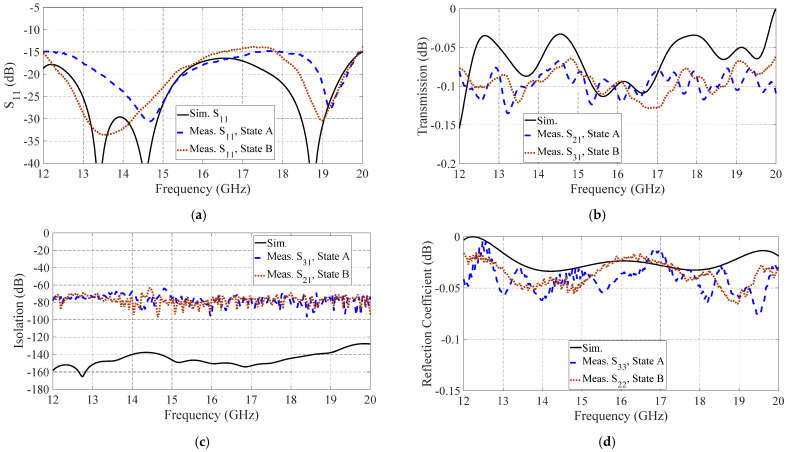
The simulated and measured (**a**) reflection coefficient (S_11_) of the pole-port, (**b**) transmission coefficient, (**c**) isolation level, and (**d**) reflection coefficient of the shorted port, for both states A and B.

The measured S-parameters of the switch are shown in Figure 10. The measurement results agreed well with the simulations. However, due to the measurement noises and mechanical tolerances, insignificant differences were detected. The measured reflection coefficient (S_11_) of the switch was less than −14 dB within the operating frequency range (12–20 GHz), representing a 50% frequency bandwidth. The maximum insertion loss was approximately 0.13 dB, while the isolation level was less than −70 dB for both states A and B. The measured isolation levels were limited by the dynamic range of the measurement equipment setup.

The proposed work was compared with previously reported microwave switches as mentioned in Table 1. The proposed switch exhibits a wide frequency bandwidth in which both return and insertion losses are low with a high isolation level compared to similar structures. The power handling capacity of the proposed switch is high compared to the others. Only Ref. [10] has a higher power handling capacity than the proposed switch, due to its lower working frequency and larger dimensions.

## 5. Conclusions

This article investigated the design and fabrication of a wideband microwave SP2T switch based on groove gap waveguide technology for Ku-band applications. The wave leakage in the sliding part of the switch was suppressed by utilizing the contactless feature of the gap waveguide technology. Therefore, manufacturing and assembling challenges were handled, and the switch was fabricated using a low-cost CNC milling technique. The measured results show that the input return loss of the switch was less than −14 dB and the insertion loss was better than 0.13 dB, while the isolation level was about 70 dB within the operating frequency bandwidth (12–20 GHz).

## Figures and Tables

**Table 1:** Comparison between published works and the proposed switch.

Type	Freq. Band	BW (%)	I.L. (dB)	Isolation (dB)	P.H. (kW)
Liquid metal [7]	K	15	0.45	>30	0.15
Rectangular waveguide [10]	X	10	0.5	>30	100
Ferrite [11]	X	6	0.5	>30	3.5
Gap waveguide [28]	X-Ku	25	0.1	>60	53
This work	Ku-K	50	0.13	>70	57.7

## Data Availability

Not applicable.
